# Qualitative care experiences of emerging adults with type 1 diabetes after transfer to adult care providers

**DOI:** 10.1111/dme.70214

**Published:** 2026-01-13

**Authors:** Daniel R. Tilden, Erin Bergner, Kashope Anifowoshe, Sydney Garretson, Nkemjika Okonkwo, Kemberlee Bonnet, Sarah S. Jaser

**Affiliations:** ^1^ Division of Endocrinology, Diabetes & Clinical Pharmacology, Department of Medicine University of Kansas Medical Center Kansas City Kansas USA; ^2^ Division of Pediatric Psychology, Department of Pediatrics Vanderbilt University Medical Center Nashville Tennessee USA; ^3^ Department of Psychology Vanderbilt University Nashville Tennessee USA

**Keywords:** emerging adults, healthcare transition, qualitative research, quality of life, social ecological model, type 1 diabetes mellitus

## Abstract

**Aims:**

Emerging adulthood is a developmental stage, which presents psychosocial, economic and academic challenges to individuals, which are further complicated for people with type 1 diabetes (T1D). While paediatric‐based care systems have developed programmes to address the needs of young adults with T1D, adult‐based systems have been slower to recognize this need. This qualitative study sought to understand the care experiences of emerging adults to identify barriers to engagement in adult‐based care systems.

**Methods:**

A total of 20 young adults with T1D (mean age 26.5 ± 4.5) participated in five focus groups. Focus groups obtained participant reflections on care received during and after transfer to adult‐based care. Data were analyzed and coded using thematic analysis and organized using the social ecological model.

**Results:**

Key themes across each social ecological model strata emerged from focus group discussions. Provider and clinic‐level factors were frequently cited as key barriers to engagement in adult‐based clinical practices. In addition, system‐ and institutional‐level gaps led young adults to express a sense that systems at all levels were poorly adapted to address their care needs. Specifically, participants noted a need for high levels of self‐advocacy to obtain needed support, difficulty navigating complex health systems and gaps in care coordination between providers and clinics as key barriers to care.

**Conclusions:**

The findings of this study highlight the importance of adapting adult care systems and providers' approaches to meet the self‐identified needs of young adults to improve engagement with care and potentially avoid adverse outcomes in this high‐risk people population.

**What's new?**

- This study examines the experience of young adults with type 1 diabetes in adult‐based care practices to understand barriers and facilitators for care after transfer from paediatric care practices.
- Gaps in self‐advocacy and difficulty in accessing support outside of the primary diabetes care provider are barriers to care engagement in adult‐based care systems.
- Improving awareness of the needs of young adults among adult‐based care systems and working to adapt these systems to facilitate accessibility for young adults may be key for improving outcomes among this population.

## 1 INTRODUCTION

**FIGURE 1:**
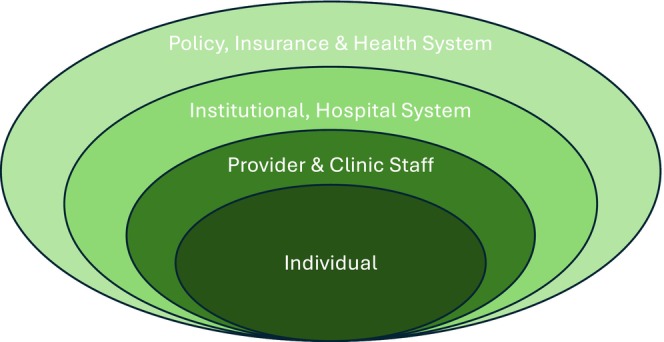
Social ecological model.^13^

The numerous biological, social and economic changes that mark the developmental stage of emerging adulthood strongly influence psychosocial and physical health outcomes among this population with type 1 diabetes (T1D).^1^, ^2^, ^3^, ^4^, ^5^, ^6^, ^7^, ^8^ While many factors influence these outcomes, the unique challenge of healthcare transition, or the move from paediatric‐to‐adult‐based care providers, has been independently associated with negative acute and chronic disease‐related complications.^9^, ^10^, ^11^ This process of transition presents emerging adults with challenges across multiple facets of their diabetes care with adult care systems.^12^ The Social Ecological Model (SEM) provides a framework for analysing the multiple levels of interactions that impact the behaviour within complex systems.^13^ Previous work examining the transition experience of adolescents and young adults with T1D has highlighted the multi‐level barriers experienced during transition, making the SEM particularly well suited to examine and explore these data (Figure 1).^14^, ^15^ These factors include individual‐level concerns about their own capacity for self care and interpersonal factors, such as leaving long‐standing relationships within paediatric care settings and establishing new relationships with providers.^16^, ^17^ In addition, system‐level barriers, such as navigating changing insurance carriers, unstable income and transportation concerns, all have been demonstrated to be factors that may inhibit successful transfers to adult care.^8^, ^14^, ^15^, ^18^, ^19^

A growing body of evidence, practice guidelines and clinical quality efforts have been initiated to help prepare young adults with diabetes for transfer to adult care.^5^, ^20^, ^21^, ^22^, ^23^ However, while complementary adult‐based care ‘receivership’ programmes to integrate young adults into adult‐based practice have also been developed, these programmes are typically more limited in scope, particularly in the United States, where complex payment, clinical and regulatory factors complicate care for this population.^21^, ^24^, ^25^, ^26^ While studies of these US‐based adult receivership programmes have demonstrated positive short‐term results, it is unclear to what extent they meet the self‐perceived needs of young adults as they move to adult care.^27^ A small number of previous studies have explored emerging adults' perceptions of adult care providers and care systems and have identified difficulty maintaining social connections and decreased access to information resources as key barriers to successful integration into adult‐based care.^15^, ^18^ These studies both were conducted in settings with universal healthcare models and fixed age of transfer. While these studies provide important data to inform the development of adult‐receivership programmes, significant variations in system‐level factors, such as variations in insurance coverage and age at transfer, may impact the generalizability of these findings.

Based on this previous work, we hypothesized that focus groups among young adults with T1D would identify both previously known and novel barriers to care across multiple levels of the SEM. To investigate this hypothesis, we conducted focus groups among young adults with T1D who had completed transfer to adult care providers with the aim of (1) understanding factors within adult‐focussed care systems, including provider and clinic level factors, which impacted transition experiences for young adults outside of structured transition programmes and (2) identifying potential adult‐based provider and clinic interventions to improve engagement and care experiences of young adults within these care delivery systems.

## 2 METHODS

### 2.1 Participants and procedures

We conducted five structured focus‐group interviews with a total of 20 young adults aged 19–35 years. Focus groups were selected to increase the range of views represented in the study and to understand shared experiences in the transition process. The study was approved by the Institutional Review Board at Vanderbilt University Medical Center (IRB #2014224), and all participants completed informed consent prior to being enrolled in the study or completing study procedures. We recruited participants using printed flyers posted in local academic and private practice endocrinology offices as well as through email and social‐media outreach from local disease advocacy organizations. Participants were eligible for the study if they were currently between the ages of 18 and 35 years, were diagnosed with T1D before the age of 18, initially were treated by a paediatric endocrinologist and were currently being treated by an adult‐based provider. All participants were required to speak English to participate in the focus groups and be available to complete the focus group and post‐survey procedures. Focus groups were scheduled at mutually agreeable times and held on multiple days, including weekends and evening hours to accommodate participant schedule conflicts.

### 2.2 Focus groups

In collaboration with the Vanderbilt Qualitative Research Core, we iteratively developed a focus‐group facilitator guide based on prior literature, the experience of the research team and guided by the SEM. The focus‐group facilitator guide had three sections of open‐ended questions. The first section included questions about young adults' experiences with adult‐based providers and clinical systems during transition, the second section focussed on their experience with transition preparation by their paediatric‐based providers, and the last section asked how their transition experience could have been improved by changing existing clinical care systems. All five focus groups were conducted via video‐teleconferencing and were facilitated by a non‐clinical member of the qualitative research team with extensive training and experience in conducting focus groups on health‐related topics. Focus groups lasted 60–90 minutes and were recorded and transcribed for analysis. The number of participants per group ranged from 2 to 7.

### 2.3 Quantitative data collection

Participants completed several surveys to provide additional quantitative data to support analysis. Prior to focus groups, participants completed a REDCap‐based demographic questionnaire including their age, gender, race, current zip code, current insurance, self‐reported most recent HbA1c, date of their most recent clinic visit and current diabetes care regimen (i.e. multiple daily injections, insulin pump use, continuous glucose monitoring and/or automated insulin delivery). After completing focus groups, participants were asked to complete the Type 1 Diabetes Distress Scale (T1‐DDS), a standardized measure of diabetes distress, and the Transition Readiness Assessment Questionnaire (TRAQ) to assess transition readiness.^28^, ^29^

### 2.4 Data analysis and statistical methods

Focus groups were transcribed and uploaded to NVivo for analysis. Techniques of thematic analysis were used to code and identify themes in the transcripts.^30^ An expert in qualitative analysis led a team of three raters to develop the initial codebook using an inductive approach based on the moderator guide and further refined based on the first two focus group transcripts. Codebook development was iterative with weekly meetings among all coders to review and refine codes, with any discrepancies in the application and definitions of codes discussed until consensus was reached, establishing inter‐rater reliability. The four raters then coded the remaining transcripts independently with ongoing meetings to develop consensus and resolve discrepancies as they arose. Counts and references to codes were monitored and saturation reached after coding of the 5th focus group. Finally, the first author, senior author and qualitative analysis leader met to review and group themes identified into SEM levels for manuscript development. Descriptive statistics were calculated using STATA version 18.0 (College Station, TX).

## 3 RESULTS

A total of 20 participants completed the study. Sample demographics are detailed in Table 1. Mean age of participants was 26.5 years (SD 4.5 years) with a mean diabetes duration of 18 years. Participants indicated high levels of transition readiness as measured by the TRAQ assessment, but also reported, on average, high levels of diabetes distress particularly in the powerlessness, eating distress, hypoglycaemia and negative social perceptions domains (Table 2).

**TABLE 1:** Participant demographics.

Participant demographics	*N* = 20
Current age [mean (SD)]	26.5 (4.5)
Diabetes duration [mean (SD)]	18 (6.8)
Gender [*n* (%)]	
Male	3 (15%)
Female	17 (85%)
Race [*n* (%)]	
Non‐Hispanic White	18 (90%)
Black	2 (10%)
Geographic location [*n* (%)]	
Rural	4 (20%)
Urban	16 (80%)
Insurance provider [*n* (%)]	
Private insurance from employer	15 (75%)
Private insurance from parent	4 (20%)
Medicare	1 (5%)
Last HbA1c [*n* (%)]	
< 52 mmol/mol (<7%)	10 (50%)
53–63 mmol/mol (7–8%)	4 (20%)
64–74 mmol/mol (8–9%)	5 (25%)
>75 mmol/mol (>9%)	1 (5%)
Time since clinic visit [*n* (%)]	
<3 months	17 (85%)
3–6 months	0
6–12 months	1 (5%)
>1 year	2 (10%)
Current diabetes technology use [*n* (%)]	
SMBG + MDI	2 (10%)
SMBG + Pump	0
CGM + MDI	4 (20%)
CGM + Pump	14 (70%)

**TABLE 2:** Participant report of diabetes distress and transition readiness.

Participant report data (*N* = 20)	Score (SD)
Transition readiness (TRAQ)^a^	
Overall score	4.5 (0.4)
Medication management	4.7 (0.4)
Appointment keeping	4.5 (0.5)
Tracking health issues	4.1 (0.7)
Talking with providers	4.7 (0.6)
Managing daily activities	4.8 (0.5)
Diabetes distress (T1‐DDS)^b^	
Overall score	2.4 (1.0)
Powerlessness	3.5 (1.5)
Management distress	1.8 (0.9)
Hypoglycaemia distress	2.4 (1.5)
Negative social perceptions	2.3 (1.4)
Eating distress	2.7 (1.0)
Physician distress	1.9 (1.5)
Friend/family distress	1.9 (1.2)

^a^ Higher scores denote increased preparation for transition.

^b^ Higher scores indicate higher distress. Scores over 2 indicate moderate distress, scores over 3 indicate high distress.

### 3.1 Major themes

Guided by the Social Ecological Model, we identified barriers and facilitators to transition from the qualitative data related to the individual, interpersonal, institutional and policy levels as summarized in Table 3.

**TABLE 3:** Key themes across levels of the social‐ecological model.

Theme	Main idea	SEM level(s)
Self‐advocacy within care settings is needed to avoid gaps in care	Participants' need to advocate with providers and clinics for preferred care. Gaps in care due to people and provider reluctance to discuss sensitive topics	Individual & interpersonal
Support from others is needed to navigate complex care systems despite desire for independence.	Care team members outside of providers are seen as key for ideal care but are often less accessible in adult care models. Continued need for education about relevant topics for young adults in clinic setting Family members remain key facilitators of care for young adults with T1D Peer support was less accessible to young adults with virtual interactions more common sources for this support	Interpersonal (provider & social support)
Gaps in clinic‐to‐clinic and system‐level care coordination hinders transition.	Limited guidance from paediatric‐based providers to assist in establishing adult‐based care. Difficulty with timely access to adult‐based provider Cost of care and insurance coverage of supplies and medications. Gaps in communication between diabetes supply providers and clinics resulting in gaps in care	Institutional/hospital & policy, insurance & health care system
Difficulty finding care that addresses care needs specific to living as a young adult with T1D.	Gaps in care due to people and provider reluctance to discuss sensitive topics Care team members outside of providers are seen as key for ideal care but are often less accessible in adult care models. Limited guidance from paediatric‐based providers to assist in establishing adult‐based care. Gaps in communication between diabetes supply providers and clinics resulting in gaps in care	Individual, interpersonal, institutional & health care system

Abbreviation: SEM, social‐ecological model.

### 3.2 Individual‐level barriers and facilitators

Participants described the importance of advocating for themselves once they had transitioned to adult care. Even among those participants who received support in other aspects of health system navigation, participants reported that they frequently were alone at clinic visits, increasing the importance of self‐advocacy. Participants reflected on needing to self‐identify concerns rather than rely on providers to elicit potential issues.

It's unfortunate that even the doctors, not just the insurance companies, you have to be your own advocate for the doctors as well because they see tons of people, and they don't know how you feel. They can run tests and stuff like that, but you have to say what you're struggling with and what you're feeling. (Participant 1; 25yo woman)

Participants acknowledged that a lack of comfort in speaking to providers contributed to delays in care and meeting with multiple providers to find one that was best for them.

Sometimes I had to switch doctors because they wouldn't give me what I wanted, and I'm the patient…and it's hard to assert yourself when you're an 18 year old to an authoritative figure like a doctor…Now I can do that as a mid‐30 year old adult, but back then, no way was I ever going to stand up to a doctor, so that caused a lot of switching and delays in care and stuff like that too. (Participant 2; 35yo F)

Participants' reluctance to discuss the many physical, mental and social changes they were facing in the context of their diabetes along with adult‐based providers not specifically identifying these as concerns also led to unaddressed areas of need:

There were no questions about alcohol, questions about sex, questions about mental health, that never got done unless there was a questionnaire that was like, do you drink? And you always put no, because you were 19 and you're not going to tell your doctor you're an underage drinker. (Participant 2; 35yo F)

This was particularly true of female participants as they faced issues such as increased sexual activity, hormonal changes and pregnancy planning.

I'm 23 now. Last year I was on a rollercoaster of blood sugars. I started my period a couple of days ago and my blood sugar was ridiculously high all week, last week, and I was so confused as to why. And then I remember that I was about to start my period. No one told me that. That's not something that's given to you in a cute little pamphlet when you're diagnosed, I don't think at any age, but no one's ever told me anything about pregnancy planning. (Participant 3; 23yo F)

### 3.3 Interpersonal/Provider barriers/facilitators

Participants reflected on the role of family and the support received from parents and siblings in taking care of their diabetes during childhood. Parents were present at doctors' visits and in communication with providers to ensure timely supply deliveries and medication refills.

My experience with prescriptions and new tech[nology] and stuff has been for most of my childhood, my mother handled it a lot. Thank goodness she was there for me and able to help me with that. She was the advocate for me up until mid‐college probably. (Participant 3; 26yo F)

After the transfer to adult care and particularly when parents were no longer involved in their child's diabetes care, participants reported difficulties finding and scheduling appointments with specialists who could provide a supportive care environment:

When I transitioned from peds to adult, I went through maybe five endocrinologists before I found a clinic where I felt safe, supported, and not judged. The sad part for me is that a lot of the clinics or the doctors that […] just threw numbers at me and then sent me on my way and didn't listen to anything I had to say. (Participant 2; 35yo F)

A common concern amongst participants was that while additional resources such as diabetes education, psychology and nutrition support were often easily accessible in paediatric care, the resources were significantly less accessible in adult care—often when they were most needed.

…it would have been such a different journey for me if when I was 18 someone had said to me, hey, you have disordered eating because you have a disease that is focused around food. If you talk to a mental health counselor or you go to therapy, or you talk to someone about this, that might help you. If my doctor had said that to me five years ago, I would be a totally different human being. I would have had a totally different experience. (Participant 3; 23yo F)

Participants also described the importance of peer support, but found these experiences were less common as they got older.

When I was growing up, I went to a summer camp for Type 1 s. I had support groups. But when you turn into an adult, unless you know someone who's a fellow Type 1, you're kind of just like on your own island. (Participant 4; 25yo F)

Participants felt that they could have better handled the many unique stressors they were facing as adults with T1D if they had access to local or online support groups of their peers. Some participants were members of Facebook groups or relied on web‐based resources that focused on T1D, which provided a sense of community and information.

I'm in a couple support groups on Facebook, and I have truly found them really helpful but also encouraging. (Participant 5; 22yo F)

I found resources online. I know Beyond Type I, their website is pretty great for adult activities, if you partake in drinking or recreational drugs, or things like that. Not from a healthcare professional, no. Just from people online. (Participant 6; 24yo F)

### 3.4 Institutional/Hospital system barriers/facilitators

Several participants noted challenges in navigating the transfer to the adult care system both from paediatric and adult care systems.

They were like, ‘Oh, you're 18 now. We can't see you anymore.’ There was no help in finding an adult endocrinologist. There was no, ‘Here's a buffer of insulin and the things you need to get you through while you find an endocrinologist.’ None of that stuff was offered. (Participant 7; 35yo F)

This was further exacerbated by frequently encountered long wait‐times for adult endocrinology visit:

Everybody had at least a six month wait by the time that I was going for adults…it took a little over 12 months to actually get into a practice. (Participant 7; 35yo F)

Delays in care also meant that participants were unable to obtain insulin and had long gaps between HbA1c tests, ranging from 6 months to several years, and even struggled with diabetes‐related complications such as DKA and seizures.

I would make decisions about my insulin dosage that I should have had more consultation with a doctor from. I shouldn't have been making those decisions. Then I had seizures that were largely my fault because I wasn't seeking the right care. (Participant 8; 31yo F)

### 3.5 Policy/Insurance/System level barriers/facilitators

Focus group participants faced significant challenges ordering necessary diabetes supplies and navigating the transition with their insurance provider even after they established with an endocrinologist. Participants particularly recalled struggling to navigate the insurance process when they were no longer on a parent's insurance plan, often preventing them from accessing necessary diabetes supplies and technology.

We heard our parents talking about the supplies and how important it is to have good insurance, but really until you are in that situation and you're going to the pharmacy or you're ordering it online, we really don't know right from wrong or what is a good price or what is our option other than what our provider is prescribing to us… Obviously now I have a handle on it, but those first couple of trips to the pharmacy is a little bit intimidating. (Participant 9; 25yo M)

The dual challenges of navigating insurance changes alongside a transition in providers often felt overwhelming for participants.

It was just going from having one doctor from the time I was diagnosed until I was 18 and then being thrown in the deep end trying to figure it out…All of a sudden, you're an adult and you're expected to understand how insurance works. I naively assumed that the level of care that I would have as an adult would be the same as the pediatric doctor who I had seen for eight years and knew my health so intimately because she diagnosed me and all this stuff. That forced me to really grow up quickly and kind of self‐educate a lot around how I need to deal with stuff and the things that I need to present to my doctors […]. (Participant 10; 31yo F)

## 4 DISCUSSION

This social ecological model‐based analysis of the qualitative focus group data allowed us to analyse emerging adults' experiences across multiple system levels to identify four central themes both within and across levels of the social ecological model. The consistent expression of a sense of adult‐care settings being poorly adapted to meet their needs—a theme across all social ecological model levels—underscores the need for tailored approaches for engaging this population in care. Additionally, our analysis identified individual and interpersonal factors as well as clinic and system‐level factors, which are barriers to transition in adult‐based care systems. These factors, such as the critical role of self‐advocacy in adult‐based care, reliance on continued support aside from standard of care visits and issues with clinic level coordination, are areas of opportunity for adult‐based providers and clinics to meet the specific needs of this population. The focussed nature of this study—on experiences in adult care systems only—adds further evidence for adult‐based providers and health systems seeking to better meet the needs of this population.^5^, ^24^, ^26^, ^31^

Using the social ecological model as our analytic framework provided several important benefits to our analysis. First, we were able to identify several key individual and interpersonal factors, which influenced their experiences of care as they entered adult care. Identifying the need to feel comfortable with advocating for their own needs and the key role of social and non‐provider support has direct implications for adult‐based care systems where self‐advocacy and autonomy are often expected by adult‐based providers. The need to address these challenges has been previously addressed in adult‐based transition interventions through case management or transition ‘navigators’.^22^, ^25^ By identifying specific challenges that may be addressed by these interventions, our data might guide refinement of these interventions by focussing on the needs of the young adults they serve.

Beyond provider‐level factors, clinic‐level factors, such as scheduling policies and identifying local clinics with a qualified provider along with insurance and system‐level factors were also barriers to care. These findings are consistent with literature identifying lapses in care in the paediatric to adult transition as a driver of adverse outcomes in this population.^9^, ^10^ A lack of endocrinologists, particularly in rural areas, was also a cause of delayed care and acute complications among participants, a finding also seen in recent literature.^9^, ^32^ The ability to address these system‐level factors is a key strength of adult‐based transfer receivership clinics which have demonstrated increases in diabetes device adoption and clinic attendance and decreases in hospitalizations in this population.^24^, ^26^ Along with providing a dedicated provider team focussed on the needs of and care for this population, these receivership clinics have the ability to streamline referrals from paediatric providers and help young adults avoid care system gaps and find a comfortable clinic‐based care setting.

The current study had several strengths. The participant sample included people with diabetes from a diverse geographical background within the United States as well as participants who were seen in both academic and community‐based practices, which is more representative of the care experience of young adults with T1D. Additionally, our study included only people who had already completed transfer to adult care and could reflect on the experience of working to establish care with an adult provider. While our sample included some participants older than traditional definitions of emerging adulthood, including this age range—some much closer to this transfer and some much farther away—we were able to capture the concerns across the process of transition as these may evolve as people with diabetes become more accustomed to adult care settings.

Along with these strengths, our study did have several limitations. Despite efforts to diversify recruitment, compared to the US population of those with T1D, study participants were significantly more likely to be White and women.^33^ While male participants generally voiced similar concerns to their female counterparts in our focus groups, it is possible that we missed factors, which may be more salient for men with T1D. More concerning, however, is the lack of representation of people from minoritized racial and ethnic groups in our sample. Given significant mistrust in healthcare institutions, which is common among those from marginalized groups, it is likely that our study did not capture or adequately address concerns, which may represent barriers or facilitators of care in this population. Finally, the focus of the moderator guide and thus subsequent discussions were primarily centred on participants' interactions with clinicians. Future work should work to both incorporate a broader sample of participants and include an interview guide focused more on understanding the role of the broader adult‐based diabetes care team on outcomes among this population.

## 5 CONCLUSION

Emerging adults with T1D are a high‐risk group who face a unique set of challenges as they navigate the transition of their care between paediatric and adult‐focussed care settings. Our study highlights several important factors across multiple domains, which are critical to young adults' success within adult care settings. In particular, fostering self‐advocacy skills prior to or shortly after transfer to adult care as well as adapting care systems—including provider, clinic and system‐level policies—where possible to understand and address the needs of this population is critical to help this population avoid short‐term complications and successfully navigate this key developmental phase.

## FUNDING INFORMATION

This work was supported by the National Institutes of Health, National Institute of Diabetes and Digestive and Kidney Diseases 3R01DK115545‐05S1 (PI: Jaser), K23DK143333 (DRT) and K12DK133995 (PI: Maahs & DiMeglio) (DRT), the Vanderbilt Diabetes Research and Training Center (P30 DK20593) (DRT, SSJ) and CTSA Award (UL1 TR002243) from the NCATS.

## CONFLICT OF INTEREST STATEMENT

DRT serves on an advisory board for Glooko, Inc. No other potential conflicts of interest relevant to this article were reported.
